# The dynamics of HER2 status in esophageal adenocarcinoma

**DOI:** 10.18632/oncotarget.25507

**Published:** 2018-06-01

**Authors:** Aafke Creemers, Eva A. Ebbing, Gerrit K.J. Hooijer, Lisanne Stap, Rajni A. Jibodh-Mulder, Susanne S. Gisbertz, Mark I. van Berge Henegouwen, Maurits L. van Montfoort, Maarten C.C.M. Hulshof, Kausilia K. Krishnadath, Martijn G.H. van Oijen, Maarten F. Bijlsma, Sybren L. Meijer, Hanneke W.M. van Laarhoven

**Affiliations:** ^1^ Center for Experimental and Molecular Medicine, Laboratory of Experimental Oncology and Radiobiology, Cancer Center Amsterdam, Academic Medical Center, Amsterdam, The Netherlands; ^2^ Department of Medical Oncology, Cancer Center Amsterdam, Academic Medical Center, Amsterdam, The Netherlands; ^3^ Department of Pathology, Cancer Center Amsterdam, Academic Medical Center, Amsterdam, The Netherlands; ^4^ Department of Surgery, Cancer Center Amsterdam, Academic Medical Center, Amsterdam, The Netherlands; ^5^ Department of Radiotherapy, Cancer Center Amsterdam, Academic Medical Center, Amsterdam, The Netherlands; ^6^ Department of Gastroenterology, Cancer Center Amsterdam, Academic Medical Center, Amsterdam, The Netherlands

**Keywords:** HER2, esophageal adenocarcinoma, dynamics, neoadjuvant therapy

## Abstract

Trastuzumab, a monoclonal antibody against HER2, has become standard of care for metastatic HER2-overexpressing esophagogastric adenocarcinoma and is currently investigated as (neo)adjuvant treatment option in HER2-positive esophagogastric adenocarcinoma. The HER2 status is commonly determined on archived material of the primary tumor. However, this status may change over the course of treatment or disease progression. The aim of this study was to assess the dynamics of HER2 status in esophageal adenocarcinoma (EAC) in patients with resectable and recurrent disease, and to determine the associations of these changes with clinical outcome. Discordance, defined as any change in HER2 status between matched biopsy and post-neoadjuvant chemoradiation therapy resection specimen (*N* = 170), or between matched resection specimen and recurrence of patients not eligible for curative treatment (*N* = 61), was determined using the standardized HER2 status scoring system. Clinically relevant positive discordance was defined as a change to HER2 positive status, as this would imply eligibility for HER2-targeted therapy. A difference in HER2 status between biopsy and resection specimen and resection specimen and metachronous recurrence was observed in 2.1% (*n* = 3) and 3.3% (*n* = 2) of the paired cases, respectively. Clinically relevant discordance was detected in 1.4% (*n* = 2) of the resectable patients and 1.6% (*n* = 1) of the patients with recurrent disease. Patients with HER2-positive status tumors before start of neoadjuvant treatment showed better overall survival, but not statistically significant. No association between HER2 status discordance and survival was found. Clinically relevant HER2 status discordance was observed and in order to prevent under-treatment of patients, the assessment of HER2 status in the metastatic setting should preferably be performed on the most recently developed lesions if the previous HER2 assessment on archival material of the primary tumor was negative.

## INTRODUCTION

Over the past years, an increase in the incidence of esophageal adenocarcinoma (EAC) has been observed in Western countries. In 2012, 52000 new cases were diagnosed, of which 53% in North America, Europe and Oceania [1]. Although new multimodality treatment strategies have been established, survival remains disappointing. For patients treated with curative intent, treatment consists of neoadjuvant chemo(radio)therapy (nCRT) followed by esophagectomy [2]. In the Netherlands, the CROSS regimen; weekly administration of paclitaxel and carboplatin for five consecutive weeks with the addition of a fractionated radiotherapy, is standard of care for patients with resectable disease. Although prognosis has increased by the addition of this neoadjuvant therapy, survival does not exceed 49 months [3]. Up until now, only two targeted therapies have been incorporated in daily practice for patients with metastatic esophagogastric cancer; (i) anti-erbb2 (HER2) targeted therapy with trastuzumab in first line treatment of HER2 overexpressing tumors, and (ii) second line anti-VEGFR2 therapy [4, 5]. Whether anti-HER2 targeted treatment strategies are of added value in patients with resectable EAC is currently being investigated in a phase I/II clinical trial (NCT02120911) [6].

Nonetheless, the question arises how to adequately select patients for HER2-targeted therapy [7]. Importantly, discordance in HER2 status may be observed between primary tumor and metastatic site(s) (synchronous discordance) and over time (metachronous discordance). The latter may either be due to changes in tumor biology over time, or to treatment effects. Little data are available on the influence of neoadjuvant chemoradiation on metachronous discordance in EAC. Hence, it is yet unknown whether to select patients for HER2 targeting therapy by determining the HER2 status on either the primary (pre-neoadjuvant treatment) tumor, the (post-neoadjuvant treatment) resection specimen, or a metachronous recurrent site.

Therefore, the aim of this study was to assess the discordance in HER2 status and HER2 protein expression between pre-treatment primary tumors and resection specimens after neoadjuvant chemoradiotherapy in a large group of patients, and between resection specimens and metachronous recurrences. In addition, we aimed to evaluate the influence of the pretreatment HER2 status on response to neoadjuvant treatment.

## RESULTS

### Study population

**Figure 1:**
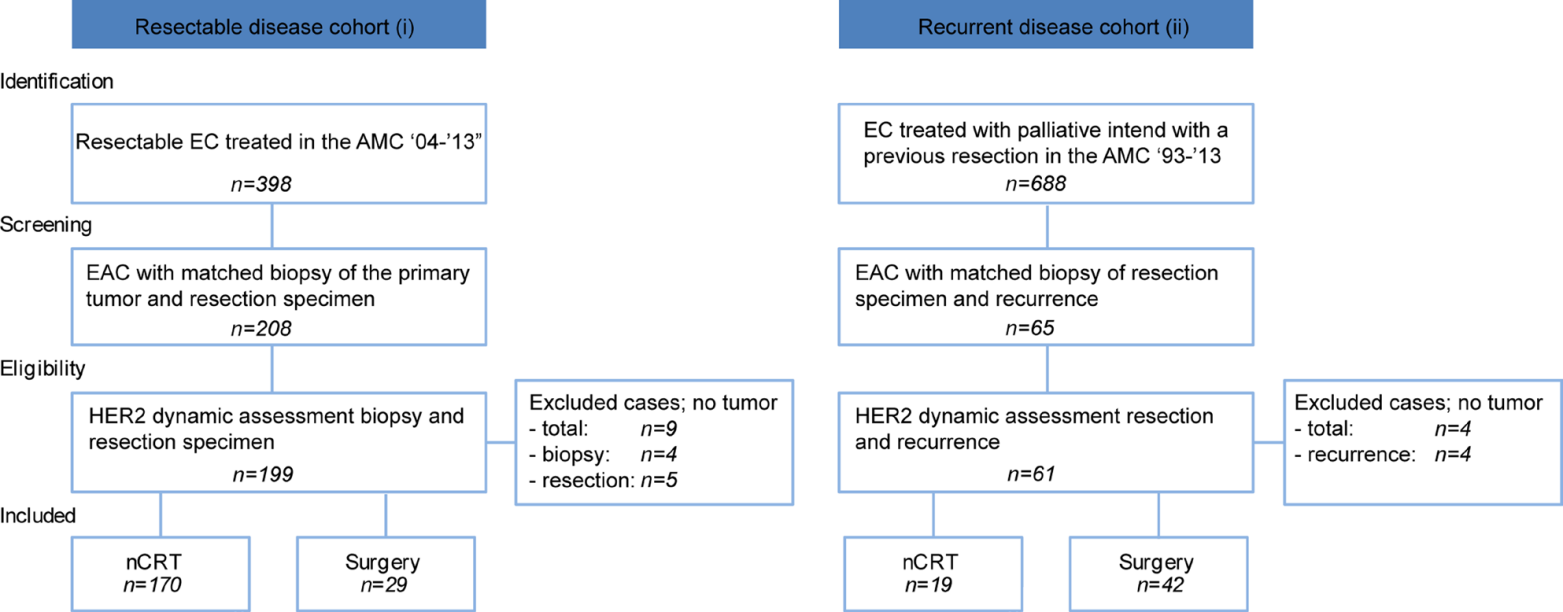
Flowchart of included patients in the resectable (i) and recurrent disease (ii) cohort

In the resectable cohort (i), all patients who had a surgical resection of the esophagus with curative intent between 2004 and May 2013 in the AMC were included (*n* = 389) (Figure 1). However, a substantial amount of cases had to be excluded due to unavailability of the biopsy of the primary tumor site or due to a histological subtype other than adenocarcinoma. In total, 179 EAC biopsies and resection specimens could be matched. Of these, four biopsies of the primary tumor site and five resection specimens had no remaining tumor tissue upon slicing of the tissue block. All 170 patients with matching biopsies and resection specimen received nCRT consisting of weekly administration of paclitaxel (50 mg per m^2^ body-surface area) and carboplatin (AUC 2 ml/min) for five consecutive weeks, and a total radiotherapy dose of 41.4 Gy in 23 fractions of 1.8 Gy. Panitumumab, an anti-EGFR monoclonal antibody, was added to the regimen in 27 (15.9%) patients as part of a randomized phase II study [8]. None of the patients received HER2 targeting therapy. Most patients were male (86%), had tumors located in the GEJ (74.7%) and had an advanced tumor stage (82.4%) (Table 1). A total of 23 of the 170 nCRT treated patients (13.5%) had complete tumor regression after nCRT (Mandard 1) and therefore no HER2 status could be assessed on the resection material (Table 1). 54.1% (*n* = 92) had a recurrence after a mean follow-up time of 45 months (standard deviation (SD) 34 months).

**Table 1:** Baseline characteristics of included patients in the resectable (i) and the recurrent disease cohort (ii)

	Curative (i)			Recurrence (ii)		
	*n* = 170	%(*n*)		*n* = 61	%(*n*)	
	HER2 negative	HER2 positive	*p*-value	HER2 negative	HER2 positive	*p*-value
	161 (94.7%)	9 (5.3%)		56 (91.8%)	5 (8.2%)	
**Mean age in years**	61.4	64.7		63.6	69.9	
**Gender**			0.223			0.804
Male	138 (85.7%)	9 (100%)		42 (75.0%)	4 (80.0%)	
Female	23 (14.3%)	0 (0%)		14 (25.0%)	1 (20.0%)	
**Location**			0.809			0.399
Proximal	1 (0.6%)	0 (0%)		0 (0%)	0 (0%)	
Mid	0 (0%)	0 (0%)		3 (5.4%)	0 (0%)	
Distal	39 (24.2%)	3 (33.3%)		34 (60.7%)	3 (60.0%)	
GEJ	121 (75.2%)	6 (66.7%)		19 (34.0%)	2 (40.0%)	
**T-stadium**			0.148			0.844
1	2 (1.2%)	1 (11.1%)		3 (5.4%)	0 (0%)	
2	25 (15.5%)	2 (22.2%)		4 (7.1%)	0 (0%)	
3	131 (81.4%)	6 (66.7%)		48 (85.7%)	5 (100%)	
4	3 (1.9%)	0 (0%)		1 (1.8%)	0 (0%)	
**N-stadium**			0.932			0.213
0	45 (28.0%)	2 (22.2%)		11 (19.6%)	3 (60.0%)	
1	100 (62.1%)	6 (66.7%)		36 (64.3%)	2 (40.0%)	
2	0 (0%)	0 (0%)		7 (12.5%)	0 (0%)	
3	16 (9.9%)	1 (11.1%)		2 (3.6%)	0 (0%)	
**pM-stadium**			NA			0.419
M0	161 (100%)	9 (100%)		51 (91.1%)	4 (80.0%)	
M1a	0 (0%)	0 (0%)		2 (3.6%)	0 (0%)	
Mx	0 (0%)	0 (0%)		3 (5.4%)	1 (20.0%)	
**Mandard grade**			0.020			NA
1	22 (13.7%)	1 (11.1%)		0 (0%)	0 (0%)	
2	27 (16.8%)	1 (11.1%)		1 (1.8%)	0 (0%)	
3	69 (42.9%)	2 (22.2%)		9 (16.1%)	1 (20.0%)	
4	35 (21.7%)	2 (22.2%)		6 (10.7%)	0 (0%)	
5	8 (5.0%)	3 (33.3%)		1 (1.8%)	0 (0%)	
Missing	0 (0%)	0 (0%)		1 (1.8%)	4 (80.0%)	
NA	0 (0%)	0 (0%)		38 (67.9%)	0 (0%)	
**Recurrence**			0.503			NA
Yes	89 (55.3%)	3 (33.3%)		56 (100%)	5 (100%)	
No	72 (44.7%)	6 (66.7%)		0 (0%)	0 (0%)	
**Type of recurrence**			NA			0.67
Locoregional	NA	NA		13 (23.2%)	2 (40.0%)	
Distant	NA	NA		23 (41.1%)	2 (40.0%)	
Locoregional and distant	NA	NA		20 (35.7%)	1 (20.0%)	

NA: not available; nCRT: neoadjuvant chemo radiotherapy

For the cohort of patients with recurrent disease (ii), 688 EC patients were selected that were treated with palliative intent, and had previously undergone resection of the primary tumor in the AMC between 1993 and 2013 (Figure 1). Of these 65 EAC resection specimen and biopsies of metachronous recurrences could be matched. Four biopsies of recurrences had no remaining tumor in the retrieved tissue sections and were excluded from further analysis. Of the resulting 61 patients, 18 patients (29.5%) were previously treated with nCRT according to the CROSS regimen, panitumumab was added to the regimen in two patients, one patient received additional hyperthermia and one patient received neoadjuvant chemotherapy without radiotherapy. None of the patients received HER2 targeting therapy or adjuvant systemic treatment. The majority of the patients were male (75.4%), had tumors located in the distal esophagus (60.7%) and pT-advanced staged disease (88.5%) (Table 1). Of the patients who received neoadjuvant treatment, none had complete tumor regression (mandard score 1). After a mean follow-up time of 30 months (SD 32), two patients were alive.

### HER2 status discordance in resectable disease

**Figure 2:**
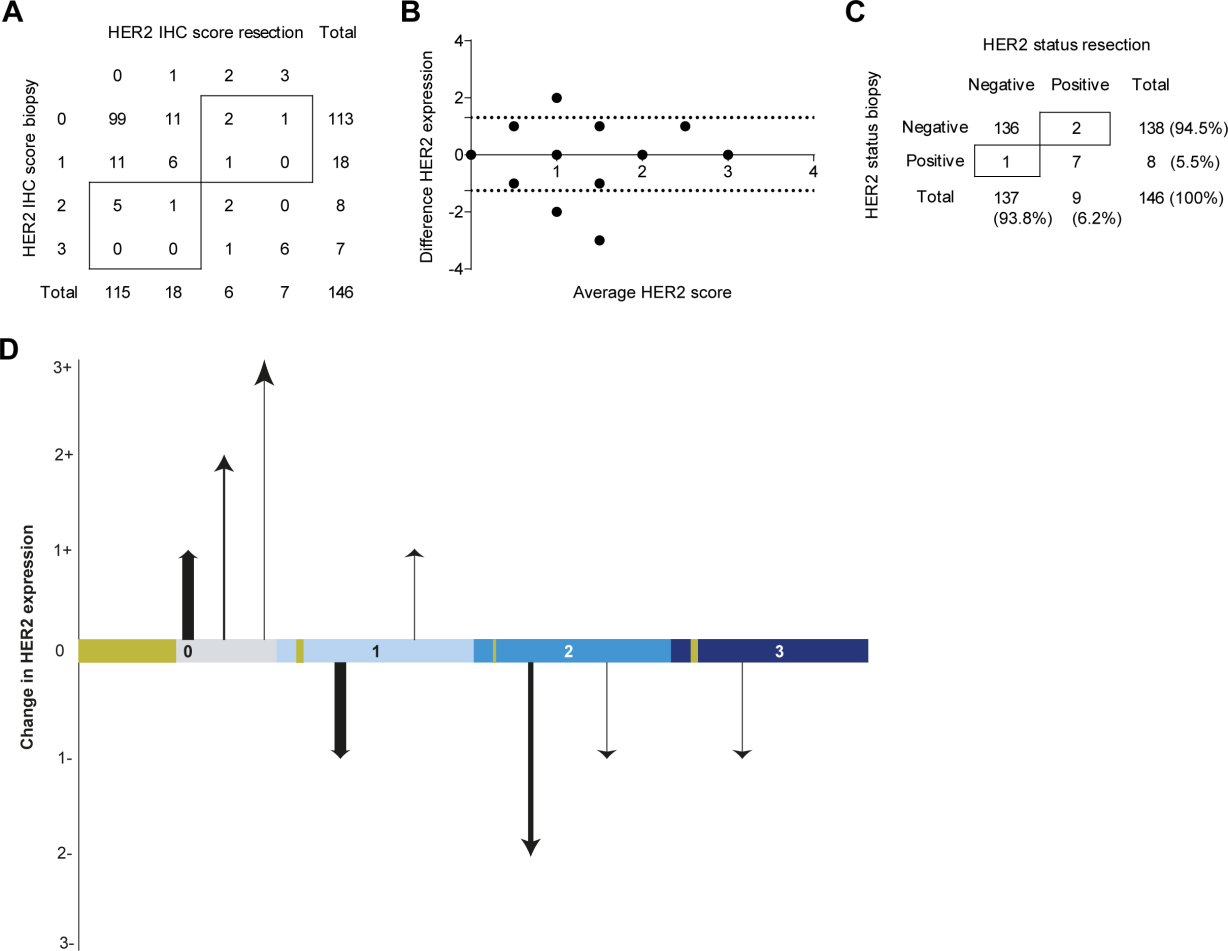
HER2 assessment of the resectable disease cohort (i) nCRT treated patients and (**A**) HER2 protein expression scores of the biopsy and resection specimen according to the scoring system of Hoffman *et al.* (**B**) The absolute observed difference in HER2 protein expression between the biopsy and resection specimen vs. the mean of both observed scores (Bland-Altman curve) [12]. The dotted lines indicate the 95% Confidence Interval (CI). (**C**) HER2 status positivity or negativity scored according to the consensus guideline; IHC and an additional SISH performed on IHC 2+ tumor material. (**D**) The change in HER2 protein expression between biopsy and resection specimen (y-axis) depicted for each score of the biopsy (x-axis). The thickness of the arrows indicate the fraction of patients undergoing this change in expression dynamics. The golden blocks on the x-axis indicate the fraction of patients not undergoing any change in expression dynamics.

HER2 protein expression assessed in pre-treatment biopsies using the Hoffman IHC scoring system demonstrated HER2-negative expression (IHC 0 or IHC 1+) in 90.0% of the cases (*n* = 153) and 4.1% (*n* = 7) HER2-positivity (IHC3+) (Figure 2A). An additional 10 biopsies (5.9%) showed equivocal HER2 protein expression levels (IHC2+), two of these biopsies showed amplification of the HER2 gene by means of SISH. Hence, 5.3% (*n* = 9) of the cases had a HER2-positive status before neoadjuvant chemoradiation therapy (Table 1). Post-treatment resection specimens showed HER2 negative protein expression (IHC 0 or IHC 1+) in 91.0% of the cases (*n* = 133), positive expression (IHC 3+) in 4.8% (*n* = 7), and 4.1% (*n* = 6) equivocal (IHC2+) HER2 expression. Of the equivocal HER2 expression cases, two showed amplification of the HER2 gene assessed with SISH. Thus, 9 out of 146 assessed post-treatment resection specimens (6.2%) had a HER2-positive status (Figure 2C). Sensitivity analyses showed no difference in HER2 status between patients treated with the CROSS regimen only, and those treated with the addition of panitumumab (*p* = 0.593).

In 146 patients the dynamics between pre-treatment biopsy and post-treatment resection specimen could be assessed. A change in HER2 status was seen in 2.1% (*n* = 3) of the cases; 1.4% (*n* = 2) had a clinically relevant positive discordance and 0.7% (*n* = 1) of the cases had negative HER2 status discordance (Figure 2 and Figure 4A). When evaluating HER2 protein expression dynamics, more positive discordance (from IHC 0 or 1+ to IHC2+ or 3+) was seen; in 2.7% of the patients treated with nCRT HER2 protein expression changed from negative to positive (Figure 2A, 2B, 2D). In contrast, 4.1% of the cases showed negative protein expression discordance after nCRT. In 77.4% (*n* = 113) of the cases no change in HER2 protein expression levels was detected.

### HER2 status discordance in recurrent disease

**Figure 3:**
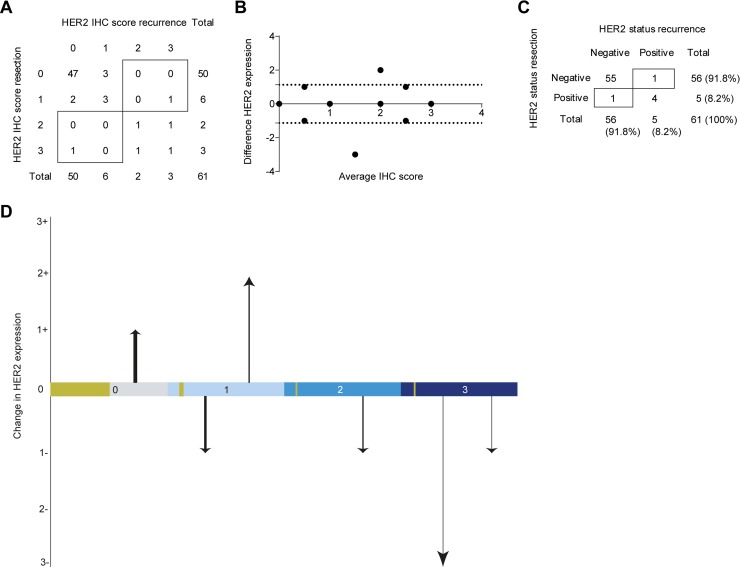
HER2 status assessment of the recurrent disease cohort (ii) (**A**) the IHC protein expression scores of the resection specimen and metastasis according to the scoring system of Hoffman *et al*. (**B**) The absolute difference in HER2 protein expression between the resection specimen and metastasis vs the mean of both observed scores (Bland-Altman curve) [12]. The dotted lines indicate the 95% Confidence Interval (CI). (**C**) HER2 status positivity or negativity scored according to the consensus guideline; IHC and an additional SISH performed on IHC 2+ tumor material. (**D**) The change in HER2 protein expression between resection specimen and metastasis (y-axis) depicted for each score of the resection specimen (x-axis). The thickness of the arrows indicate the fraction of patients undergoing this change in expression dynamics. The golden blocks on the x-axis indicate the fraction of patients not undergoing any change in expression dynamics.

**Figure 4:**
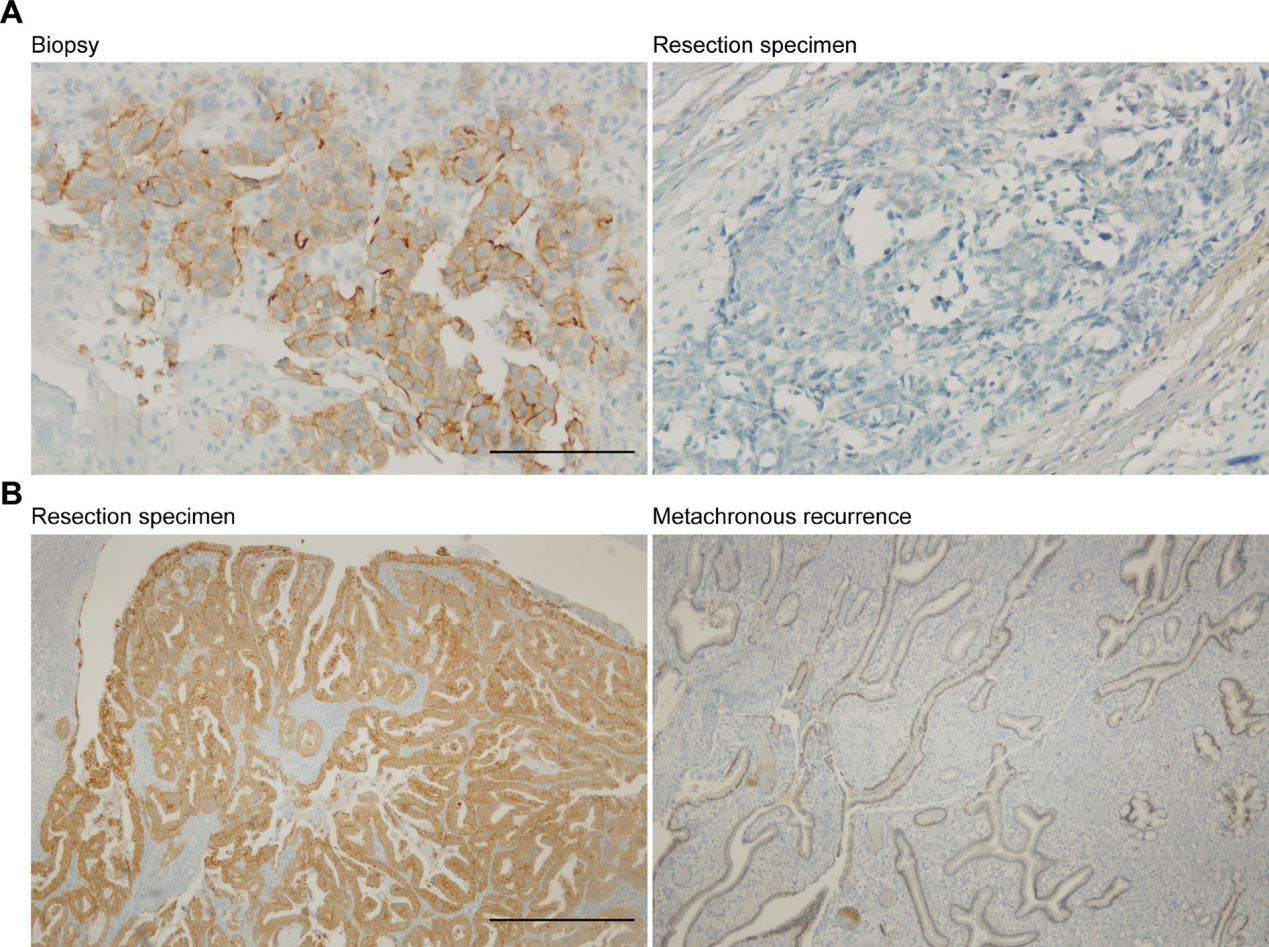
Example of discordant cases (**A**) Discordance between primary tumor biopsy and post-treatment resection specimen Scale bar: 200 μm (i). (**B**) Discordance between primary tumor resection specimen and metachronous recurrence (ii). Scale bar: 200 μm.

HER2 expression was negative in 91.8% (*n* = 56) of the resection specimens in the cohort with recurrent EAC (IHC 0 and IHC 1+) (Table 1). Three resection specimens were scored HER2 protein expression IHC 3+ and two (3.3%) resection specimens showed equivocal HER2 protein expression (IHC 2+), both had HER2 gene amplification detected by SISH (Figure 3A, 3C). Thus, 8.2% (*n* = 5) of the resection specimens had a HER2 positive status (Table 1). Of the metachronous recurrences 8.2% (*n* = 5) were HER2 positive; 3 recurrences were scored IHC 3+ and two tumors showed equivocal HER2 protein expression (IHC 2+) with amplification of the HER2 gene assessed by SISH. HER2 status discordance between primary tumor and metastasis was seen in 3.3% (*n* = 2) of cases (Figure 3C and Figure 4B). Both clinically relevant positive, as well as negative discordance HER2 status was detected in 1.6% (*n* = 1) of the cases. None of the patients with a discordant HER2 status received neoadjuvant chemoradiotherapy. HER2 protein expression dynamics were detected in 14.8% (*n* = 9) of the patients between resection specimen and metachronous recurrence (Figure 3A–3C). As amplification of the HER2 gene was observed in all HER2 IHC2+ scoring resection specimens and recurrences, based on HER2 protein expression correspondingly 1.6% (*n* = 1) positive and negative discordance was seen.

### Correlation between HER2 status, clinicopathological parameters, and survival

In the patients with resectable disease, comparing proximal, mid or distal EAC with GEJ tumors, HER2 positive status of the pre-treatment primary tumor biopsy showed no significant association with location of the primary tumor (Table 2.1.1.). Also, a HER2-positive status of the pre-treatment biopsy was not significantly related to T-stage. In multivariate logistic regression analysis including location of the primary tumor and pT-stage, none of the clinicopathological parameters showed a significant association with the HER2 status assessed in pre-treatment biopsies of the primary tumor (Table 2.1.2.). When assessing the influence of pre-treatment HER2 status on Mandard score and recurrence, no statistically significant association was found in univariate analyses (Table 2.2.1). However, although not statistically significant, a higher odds ratio was seen for high Mandard score in patients with a pre-treatment HER2 positive status (OR 3.430, 95% CI (0.880–13.370), *p* = 0.076). Moreover, 73.3% of the EACs with a negative HER2 status determined in pre-treatment biopsies had a low Mandard score (1–3) vs. 44.4% of the pre-treatment HER2-positive status EAC biopsies. No significant association between pre-treatment HER2 status and survival was found, however, a trend towards poorer prognosis in HER2-negative status patients was seen (Supplementary Figure 1A). Moreover, this trend was also seen in multivariate Cox-regression analysis including T-stage, Mandard score and pre-treatment HER2 status Supplementary Figure 1B).

**Table 2:** Regression analysis

**2.1.1**																						
**Univariate analyses**	**Dependent variable pre-treatment biopsy HER2 status**																					
	HER2 negative							HER2 positive							95% CI							
	*n* = 161							*n* = 9				OR			lower limit			upper limit			*p*-value	
**EC**	40 (24.8%)							3 (33.3%)				0.661			0.158			2.766			0.571	
**T-stage T1-2**	27 (16.8%)							3 (33.3%)				0.403			0.095			1.712			0.218	
	**Dependent variable mandard score low vs. high**																					
	Mandard low							Mandard high							95% CI							
	*n* = 122							n= 48				OR			lower limit			upper limit			*p*-value	
**HER2 positive**	4 (3.3%)							5 (10.4%)				3.43			0.88			13.37			0.076	
	**Dependent variable recurrence**																					
	yes							no							95% CI							
	*n* = 95							*n* = 75				OR			lower limit			upper limit			*p*-value	
**HER2 positive**	3 (3.2%)							6 (8.0%)				1.618			0.391			6.696			0.507	
**2.1.2**																					
**Multivariate analyses**		**Dependent variable HER2 status pre-treatment biopsy**																			
							95% CI														
		OR					Lower limit							Upper limit						Sig.	
**T-stage T1-2**		0.426					0.096							1.880						0.260	
**T3-4**																					
**EC**		0.783					0.179							3.431						0.746	
**GEJ**																					

**2.2.1**																		
**Univariate analyses**						**Dependent variable discordance HER2 status**												
									95% CI									
						OR			lower limit		upper limit					*p*-value		
**Location EC vs. GEJ**						0.168			0.015		1.911					0.151		
**T-stage T1-2 vs. T3-4**						0.383			0.033		4.405					0.441		
**Mandard score low vs. high**						4.217			0.373		47.712					0.245		
						**Dependent variable recurrence**												
			yes			no					95% CI							
			*n* = 83			*n* = 63			OR		lower limit					upper limit		
**HER2 discordance**			1 (1.2%)			2 (3.2%)			0.372		0.033					4.196		
**2.2.2**																
**Multivariate analyses**				**Dependent variable HER2 status discordance**												
					95% CI											
				OR	Lower limit					Upper limit			Sig.			
**T-stage T1-2**				0.327	0.022					4.752			0.413			
**T3-4**																
**Location EC**				0.170	0.014					2.029			0.161			
**GEJ**																
**Mandard score low**				6.129	0.433					86.751			0.180			
**High**																

2.1.1. Univariate logistic regression of the influence of the location of the primary tumor and T-stage on the pre-treatment HER2 status, and the influence of the HER2 status of pre-treatment biopsies on the Mandard score and recurrence. 2.1.2. Multivariate analysis of the pre-treatment HER2 status on the location of the tumor and T-stage. 2.2.1. Univariate logistic regression of the influence of the location of the primary tumor, T-stage and Mandard score on the discordance in HER2 status between pre-treament biopsy and resection specimen post-chemoradiation therapy, and the influence of this HER2 status discordance on recurrence. 2.2.2. Multivariate logistic regression analysis of the influence of T-stage, location of the primary tumor and Mandard score on discordance in HER2 status between pre-treatment biopsies and resection specimen post-chemoradiation therapy.

Discordance in HER2 status between pre-treatment biopsies and post-treatment resection specimen was not associated with the location of the primary tumor, T-stage, Mandard score or recurrence (Table 2.2.1. and 2.2.2.). Furthermore, HER2 status discordance between pre-treatment biopsy and post-treatment resection specimen was not significantly associated with either OS or DFS.

## DISCUSSION

This is the largest study to investigate HER2 status discordance between pre-treatment biopsies and post-neoadjuvant chemoradiation therapy resection specimens in EAC. We identify HER2 status discordance in 2.1% of the resectable cases, of which 1.4% positive and 0.7% negative discordances were observed. Importantly, positive discordance could be clinically relevant, as in principle these patients are eligible for HER2 targeting therapy, either in the context of clinical studies in the adjuvant setting, or as standard of care if they were to develop metastatic disease.

In patients with recurrent disease we identified 3.3% discordance in HER2 status; 1.6% positive and 1.6% negative discordance. The positive discordance in our study is lower than previously reported in our meta-analysis, where 5% positive discordance was observed. However, the majority of the included cases in the meta-analysis were synchronous metastases in lymph nodes, rather than metachronous metastases, and non-neoadjuvantly treated patients [7]. Given these low disconcordance rates, assessment of HER2 status on archived material may be a valid approach if recurrent lesions are not accessible for biopsy sampling. Also, it should be noted that there have been reports showing that taking of biopsies may be associated with tumor growth and malignancy [9]. However, to the best of our knowledge, this has not been described for esophageal cancer, especially not in the clinical setting. However, our data do suggest that re-assessment of HER2 status in patients with metachronous recurrent disease with previously HER2 status negative tumors may be relevant to prevent under-treatment of patients. Previous studies have demonstrated a survival benefit for HER2 positive patients of treatment with HER2 targeting therapy added to a doublet backbone of a platinum and capecitabine/5-FU, with manageable side effects [10]. Therefore, if patients are fit and willing to undergo cytotoxic treatment, HER2 status should be investigated to assess whether trastuzumab should be added to the treatment regimen. Of note, however, in breast cancer patients with a positive discordant HER2 status treated with HER2 targeted therapy, a reduced survival was observed compared to patients with concordant HER2 positive status [11]. The implications for HER2 targeting treatment of HER2 status discordance in EAC has not been addressed. Moreover, due to the retrospective nature of the here described cohort, this question should be investigated in future clinical studies.

Overall, HER2 status positivity in our cohort was lower than previously reported by other groups [12–15]. Earlier research has shown that differences in reported HER2 status might be the result of different antibodies and probes used when assessing HER2 status [16]. Here we have used the HER2 monoclonal antibody SP3, the most commonly used antibody in clinical practice. It should be noted that in less recent papers, a HER2-positive status was defined as HER2 protein expression IHC score 3+ and 2+, without additional *in situ* hybridization to assess HER2 gene amplification, thus leading to a higher number of HER2 positive cases [17–19]. Several studies have demonstrated the necessity of taking several biopsies of the primary tumor to account for intratumoral heterogeneity and to adequately determine a HER2-positive status [20]. As we used a retrospective cohort, multiple biopsies of the primary, or metastatic tumor site were unfortunately not available.

We observed a relatively low number of the HER2 protein expression IHC 2+ scoring tumors to have an amplification of the *HER2/ERBB2* gene. In a large tissue microarray study including 1040 gastro-esophageal cancer cases, HER2 protein overexpression was not associated with *HER2* gene amplification in 10% of the cases. Although in general both SISH and FISH show high sensitivity for HER2 gene amplification, SISH is easier to interpret, and is therefore more often applied in daily clinical practice. Yet, Rauser and colleagues demonstrated that new FISH techniques show more accurate detection of HER2 gene amplification [21]. As we used SISH, a higher number of HER2 positive cases might have been detected if these new FISH techniques were used. Further exploration of the most adequate *in situ* hybridization method to select eligible patients for HER2 targeted therapy is required.

However, it is not yet clarified if *HER2* amplification by itself is required for response to anti-HER2 therapy. Subgroup analyses in the ToGA trial demonstrated that HER2 protein expression negative (IHC 0 or 1+) tumors with amplification of the *HER2* gene assessed by ISH, had no survival benefit of the addition of trastuzumab to the standard chemotherapy regimen [5]. As anti-HER2 targeted therapy is of course directed against the HER2 protein on the cell surface, it is well possible that patients with tumors with a HER2 IHC protein expression score of 2+ without gene amplification may derive benefit from HER2 targeted therapy.

When investigating the association between the HER2 status in pre-treatment biopsies and OS, no significantly association with OS was observed, although a trend towards better survival was seen in HER2-positive status patients. On the contrary, a non-significant higher odds ratio for a high Mandard score was seen in HER2-positive patients. These apparently potentially contradictory results might be explained by the recent finding in EAC that HER2 expression is required to maintain an epithelial phenotype [22]. Loss of HER2 was demonstrated to result in a more mesenchymal phenotype, in which cells obtain an enhanced migratory capacity, resulting in a more aggressive tumor cell behavior, and the development of metastatic disease, contributing to a poor patient survival [23, 24]. Therefore, despite a poorer response to neoadjuvant treatment, a HER2 positive status may be associated with a more consistent epithelial phenotype, resulting in a better survival for HER2 positive patients.

## METHODS

### Study cohort

The study cohort was established by systematically searching medical records of the Academic Medical Center, a national referral center for esophagogastric cancer, including cases between 2004 and May 2013. Two cohorts were established: (i) resectable disease; patients with histologically proven EAC -including the gastric junction (GEJ) defined as Siewert type I and II- treated with curative intent with chemoradiation followed by resection of whom a biopsy of the primary tumor site and the corresponding resection specimen were available; (ii) recurrent disease; patients with histologically proven recurrent EAC, not eligible for curative treatment, of whom a resection specimen and corresponding metachronous recurrence could be obtained. Patient characteristics were retrieved from the medical records by a trained physician using a standardized extraction form. Extracted data included location of primary tumor, TNM stage based on the pathological report of the resection (pTNM), Mandard score, treatment received, date of recurrence, and survival [25]. Both the histological subtype and response to therapy were assessed by a trained pathologist. None of the patients received anti-HER2 targeting therapy. The formalin fixed paraffin-embedded (FFPE) material was retrieved in compliance with the Helsinki Declaration of 1975 [26].

### HER2 testing method

**Figure 5:**
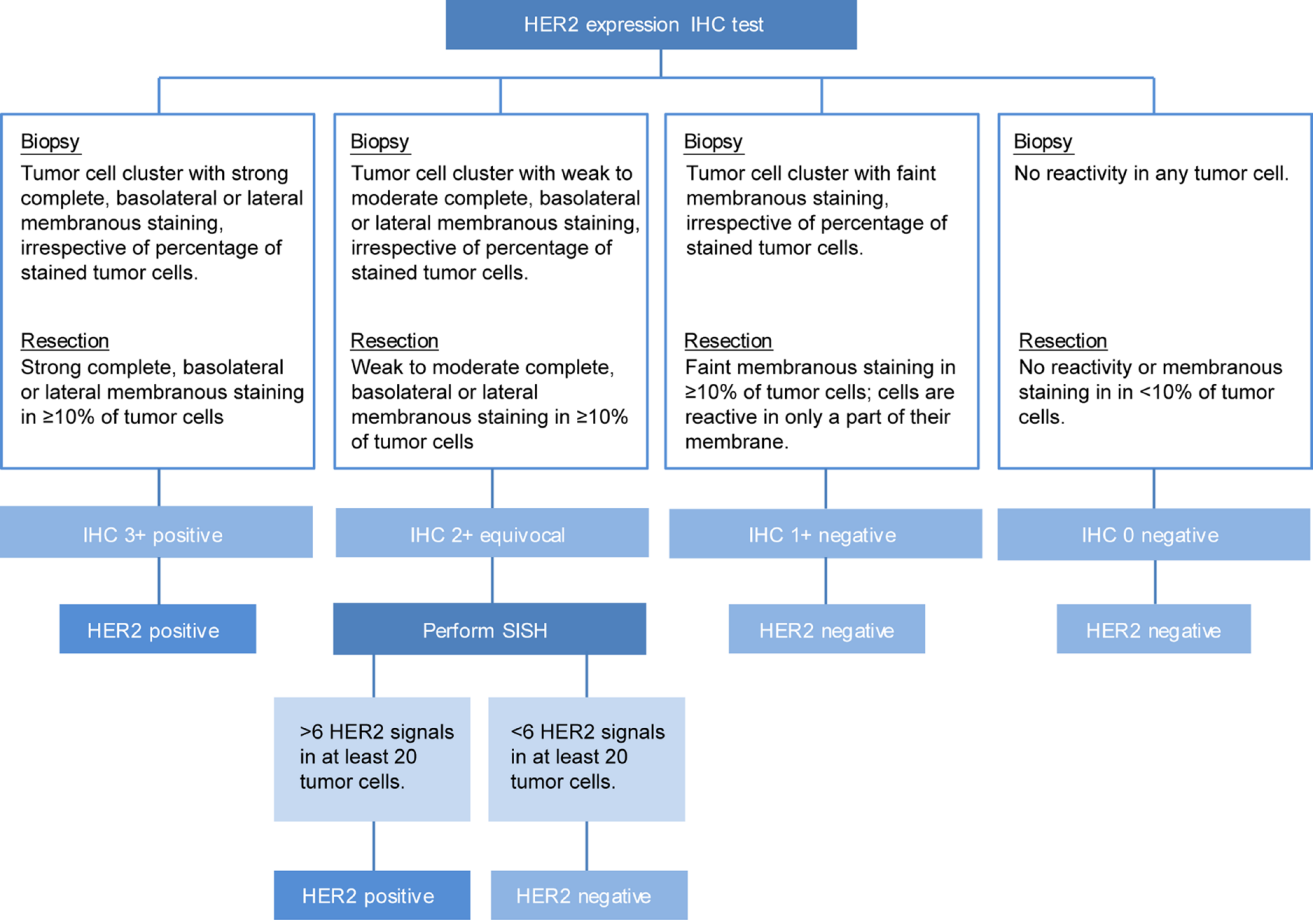
Flowchart of applied HER2 assessment for FFPE slides of biopsies, resection specimen and recurrences based on the ASCO guideline 2016 [29]

4 μm FFPE sections were cut using a microtome (Thermo Scientific Microm HM 340, Walldorf, Germany). A consecutive hematoxylin and eosin staining was performed to check for vital tumor content. The anti-HER-2/c-erB-2/neu (clone SP3) antibody (Thermo Fisher Scientific, Lafayette, CO, USA) was used to assess HER2 protein expression. The paired slides of the biopsy and resection specimen (i), or resection specimen and recurrences (ii), were simultaneously stained on an automated immunostainer (Benchmark XT Ventana, Tucson, USA). The slides were scored by a certified pathologist according to the current gold standard, using the Hoffman scoring system to score HER2 protein expression (Figure 5) [27]. Tumors scoring IHC 0 or 1+ were defined as HER2-negative, IHC3+ scoring tumors were defined HER2-positive. If HER2 expression was scored equivocally (IHC 2+), an additional SISH was performed using the INFORM HER2 Dual ISH DNA Probe assay and visualized with the ultraVIEW SISH detection kit using a Ventana Benchmark XT platform (Ventana, Tucson, Arizona, USA). Amplification of the HER2 gene was defined as more than 6 HER2 signals in at least 20 tumor cells. Those IHC2+ scoring tumors with an amplification of the HER2 gene were also defined to have a HER2-positive status. Two types of metachronous discordance in HER2 status were defined: 1. positive discordance; a change from HER2-negative to -positive status; 2. negative discordance; a change from HER2-positive to -negative status. As patients with HER2-positive status tumors are eligible for anti-HER2 therapy, a positive discordant HER2 status was seen as a clinically relevant discordance. In addition, we also assessed HER2 dynamics based solely on HER2 protein expression. In case of HER2 assessment based on protein expression, overexpression was defined as HER2 IHC score 2+ and 3+. Conversely, a HER2 protein IHC score of 0+ or 1+ was defined as HER2-negative protein expression. When assessing HER2 protein expression dynamics, positive discordance was defined as a change from IHC0 or 1+ to IHC 2+ or 3+, and negative discordance as a change from IHC 2+ or 3+ to IHC0 or 1+.

### Statistical analysis

Differences in clinicopathological variables between patients with HER2-positive status tumors and HER2-negative status tumors were assessed using the Pearson's chi-square test. Dynamics in HER2 status and HER2 protein expression were evaluated using cross tabulations. Sensitivity analyses were performed on those patients receiving panitumumab treatment in addition to chemoradiation in the context of a clinical trial [8]. In addition, a Bland-Altman curve was plotted to visualize the mean difference in HER2 protein expression between both pre-treatment biopsies and resection specimens post-chemoradiation therapy, and resection specimens and metachronous recurrences [28]. The influence of clinicopathological variables on the dynamics of the HER2 status was evaluated with univariate and multivariate logistic regression analysis. Clinically relevant variables, defined as a *p*-value of *p* < 0.3, were included in the multivariate logistic regression model. Furthermore, we assessed the influence of the location of the tumor and T-stage on pre-treatment HER2 status. Also, the influence of pre-treatment HER2 status on response to treatment (Mandard score) and recurrence was determined with univariate logistic regression analysis [25]. A low Mandard score was defined as Mandard score 1–3 vs. a high Mandard score 4–5. Survival analyses for the dynamics in HER2 status and pre-treatment HER2 status was performed using Kaplan-Meier and multivariable Cox proportional hazard regression analysis, including variables with a *p* < 0.3. Overall survival (OS) time was computed from the date of diagnosis to the date of death, censored for a non-cancer related cause of death, surviving patients were censored at the date of last follow-up. Disease free survival (DFS) was defined as the time between the resection of the primary tumor and disease recurrence. Statistical analyses were performed in IBM SPSS statistics 24.0 (IBM, Armonk, NY, USA), a *p*-value of *p* < 0.05 was regarded statistically significant. The Bland-Altman analysis was performed and plotted using GraphPad Prism 7 (GraphPad Software, San Diego, CA, USA).

## CONCLUSIONS

In our cohort of patients with resectable disease, a discordance in HER2 status between biopsy and resection specimen was observed in 2.1% of patients that received neoadjuvant treatment. A clinically relevant positive discordance was seen in a subset of patients (1.4%), generating a possible therapeutic window for HER2 targeting therapy. Patients with pre-treatment HER2-positive tumors showed a trend towards better overall survival, but no association between HER2 discordance and survival was seen. Discordance between primary tumor and paired metachronous recurrences was detected in 3.1% of the cases, 1.6% of these cases with recurrent disease demonstrated a potentially clinically relevant positive discordance. Although the number of discordant cases was low, to prevent undertreatment, we advocate that HER2 status should be assessed on the most recently developed lesions if HER2 assessment in previous biopsies was negative.

## SUPPLEMENTARY MATERIALS FIGURES AND TABLES



## Abbreviations

EAC: Esophageal adenocarcinoma
DFS: disease free survival
EGFR: Epidermal growth factor receptor
Erb-b2/HER/ERBB: human epidermal growth factor receptor
FFPE: formalin fixed paraffin-embedded
GEJ: gastric junction
nCRT: neoadjuvant chemoradiotherapy
OS: overall survival
(p)TNM: (pathological) tumor, nodes
SISH: silver *in situ* hybridization
VEGFR: Vascular Endothelial Growth Factor
