# Supplementary material for: The dynamics of HER2 status in esophageal adenocarcinoma
Source: Oncotarget. 2018 Jun 1;9(42):26787–99. doi: 10.18632/oncotarget.25507 (PMC6003553; doi:10.18632/oncotarget.25507)
Supplement: Supplementary file 1 [file oncotarget-09-26787-s001.pdf]

The dynamics of HER2 status in esophageal adenocarcinoma

SUPPLEMENTARY MATERIALS

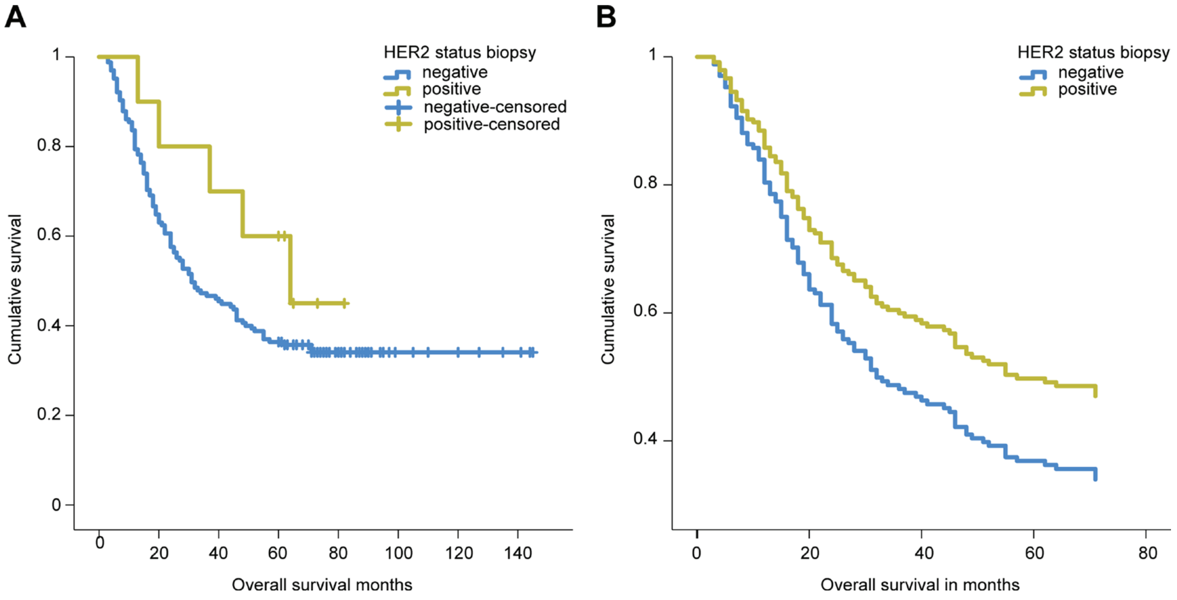

Supplementary Figure 1: (**A**) Kaplan Meier survival analysis of HER2 status in pre-neoadjuvant treatment biopsies of the primary tumor on overall survival. (**B**) Cox regression analysis of pre-treatment HER2 status of the primary tumor and overall survival, including mandard score and T-stage.
